# Plate-based diversity subset screening generation 2: an improved paradigm for high-throughput screening of large compound files

**DOI:** 10.1007/s11030-016-9692-9

**Published:** 2016-09-08

**Authors:** Andrew S. Bell, Joseph Bradley, Jeremy R. Everett, Jens Loesel, David McLoughlin, James Mills, Marie-Claire Peakman, Robert E. Sharp, Christine Williams, Hongyao Zhu

**Affiliations:** 1 Pfizer Worldwide Research and Development, Groton, CT USA; 2 Pfizer Worldwide Research & Development, Sandwich, Kent UK; 3 Imperial College, London, UK; 4 Scitegrity Ltd, Discovery Park, Sandwich, Kent UK; 5 University of Greenwich, Chatham Maritime, Kent UK; 6 JL Consulting, Deal, Kent UK; 7 Eli Lilly & Company, Indianapolis, IN USA; 8 Sandexis Medicinal Chemistry Ltd, Canterbury, Kent UK; 9 RES Consulting, San Francisco Bay, CA USA; 10 Ipsen, Slough, Berkshire UK

**Keywords:** Rule of 40, Ro40, High-throughput screening (HTS), Plate-based, Diversity, Subset, Screening file, 2nd Generation, Lead discovery

## Abstract

High-throughput screening (HTS) is an effective method for lead and probe discovery that is widely used in industry and academia to identify novel chemical matter and to initiate the drug discovery process. However, HTS can be time consuming and costly and the use of subsets as an efficient alternative to screening entire compound collections has been investigated. Subsets may be selected on the basis of chemical diversity, molecular properties, biological activity diversity or biological target focus. Previously, we described a novel form of subset screening: plate-based diversity subset (PBDS) screening, in which the screening subset is constructed by plate selection (rather than individual compound cherry-picking), using algorithms that select for compound quality and chemical diversity on a plate basis. In this paper, we describe a second-generation approach to the construction of an updated subset: PBDS2, using both plate and individual compound selection, that has an improved coverage of the chemical space of the screening file, whilst only selecting the same number of plates for screening. We describe the validation of PBDS2 and its successful use in hit and lead discovery. PBDS2 screening became the default mode of singleton (one compound per well) HTS for lead discovery in Pfizer.

**Electronic supplementary material:**

The online version of this article (doi:10.1007/s11030-016-9692-9) contains supplementary material, which is available to authorized users.

## Introduction

The pharmaceutical industry is undergoing a period of significant change that is characterized by site closures, reduction in research and development (R&D) operations, an increased use of outsourcing as opposed to in-house operations, increased collaboration with academic partners and an increased focus on biological agents as well as small molecules [1–3]. These changes are driven by the disconnect between the rising investments in drug discovery R&D and the decline in new drug outputs [4]. This poor productivity has been attributed to project attrition, which has been especially acute in Phase II of the development process [5], where project survival as low as 10 % has been recorded. Many proposals for improving the current situation have been proposed [6–11], and although a significant increase in new drug approvals has been reported over the past two years [12, 13], all elements of the drug discovery process are nevertheless being scrutinized. An operation such as high-throughput screening (HTS) [14–16] not only requires expensive hardware for screening itself, but also requires significant investments in the compound collection, the material management processes to create, store and access the file, the informatics system to underpin all these efforts and, of course, the expert scientists to operate it. In large companies, it is common for the screening file to comprise millions of compounds [17, 18]. An HTS against such a file could take several months to screen and cost a considerable amount in terms of reagents, consumables and staff time. It is therefore natural for drug discovery scientists to investigate faster and cheaper options for lead discovery that might have similar success rates with greater efficiency. Several approaches have been explored including virtual screening [19–22], where the screening event is entirely *in silico*, or fragment screening [23–26] where the low molecular weight of the fragment compounds used for lead discovery means that a larger percentage of the chemical space can be covered with far fewer compounds than for a conventional screening file of larger compounds. Virtual screening has the advantages of speed and low costs; however, the probable success of virtual screening against a novel target, or against a target for which only an apo structure is available [27], will be low due to the difficulties of predicting ligand–target interactions in the absence of structural information on that complex. Fragment screening is highly effective and becoming increasingly so, even against novel targets, but suffers from the drawbacks of generally finding weak binding hits and the fact that hit follow-up is hindered unless supported by structural biology information to guide the chemist.

Much effort has also gone into improving the efficiency of HTS approaches themselves including the use of (i) diverse subset screening [28, 29], where a portion of the file is selected to represent the entire file on the basis of chemical diversity [30]; (ii) targeted subset approaches where a portion of the file is selected on the basis of the predicted increased likelihood of finding active compounds against a specific target or target gene family [31, 32] using existing knowledge and (iii) compressed screening [33, 34] where compounds are mixed together into pools such that far fewer wells need to be screened, depending on the degree of compression. These approaches have the efficiency benefit of screening fewer wells. However, there are potential drawbacks to all these approaches in terms of success rates. Diverse subset approaches can suffer from the issue of selecting for bizarre outlier compounds, unless care is taken with the selection algorithm, and will always be inferior to screening the full file, because potentially active compounds are being deselected for screening. Targeted subset approaches will only be as good as the knowledge base used to make the selection and will suffer from the additional issue of failing to find hits that are in novel or in non-obvious areas of chemical space. Compressed screening approaches suffer from the combined problems of increased compound load, directly proportional to the number of compounds in each well, and of potential compound interactions in the mixtures, which can lead to high false-positive rates and potentially false negatives as well.

In order to find new solutions to the problem of increasing HTS efficiency, we introduced a novel plate-based diversity subset (PBDS) for singleton (one compound per well) HTS in Pfizer in 2006 [35] and Novartis reported on similar implementations in 2007 [36] and 2009 [37]. The PBDS was constructed by a novel process that (i) selected only high-quality screening plates that were Rule of 40 (Ro40) [35] compliant, (ii) used a random-based plate selection process to avoid false minima in the optimization of plate selection and (iii) was programmed to provide a double coverage of >95 % of the BCUT [38] chemical space of the 2006 Pfizer screening file in a minimal number of plates (around one-eighth the number of screening plates of the entire file). We now report on the design, validation and successful use of an improved, second-generation, plate-based diversity subset (PBDS2). An update to the first subset selection was necessary, as the original PBDS had become out of date, with the significant growth of the Pfizer screening file between 2006 and 2009. However, in creating PBDS2, we took the opportunity to not only sample new chemical space, but also to enhance significantly the selection methodology and design, such that the new PBDS2 provides a double coverage of 100 % of the BCUT chemical space of the enlarged screening file, whilst only using the same number of plates as the original PBDS. PBDS2 became the standard method of singleton-HTS-based lead discovery in Pfizer after its introduction in 2009 and here we describe its design, implementation and initial successes.

## Results

### Construction of the plate-based diversity subset generation 2 (PBDS2)

#### The Pfizer screening file

The Pfizer screening file in the fourth quarter of 2008 comprised ca 10,198 plates (384-well) and ca 3.7 million compounds. The plates were processed using the following four steps prior to the selection of plates for the PBDS2. As in the original PBDS construction [35], the Global Diverse Representative Subset (GDRS) filters [28] were applied to flag and then remove compounds with undesirable substructures or properties. Similarly, duplicate compounds and compounds with any structural ambiguity were also removed.

1. A list of plate identifiers (IDs) was processed to generate compound IDs for each compound on each screening plate.
2. Compound IDs were translated into chemical structures (SMILES, Simplified Molecular-Input Line-Entry System).
3. SMILES were checked for uniqueness.
4. Structural filters [Global Diverse Representative Subset (GDRS)] [28, 35] were applied to identify and computationally ‘remove’ compounds with unwanted (reactive or otherwise bad) functionality.

The 3,263,170 remaining, well-defined, non-duplicate, filter-pass compounds (89 % of the total set) were used for the analysis and were processed in the following way:

1. Rule of 5 (Ro5) [39] properties for each compound were calculated and summarized on a plate basis.
2. The number of unique compounds on each plate was determined (compounds that had failed the GDRS filters were not counted).
3. Stricter structural filters [35, 40] were applied on each individual compound and the number of compounds free of these structural alerts on each plate was calculated.
4. Five-dimensional BCUT [38] coordinates were calculated for each individual compound and BCUT summaries generated for each plate.
5. Each compound was classified as being derived via parallel or library chemistry (via its library ID) or through non-library synthesis (absence of library ID) and these classifications were summed up for each plate.

#### Screening plate quality for PBDS2 plate selection:

The 384-well screening plates selected for the original PBDS [35] were all compliant with the Rule of 40 (Ro40). This was to ensure that only plates of the highest quality were included into this critical subset, which was the mainstay of singleton screening in Pfizer from 2006. Each screening plate contains up to 360 test compounds and has the remaining 24 positions reserved for control compounds. The Ro40 [35] specifies that in order to be compliant, a 384-well plate must have

1. more than 200 unique test compounds,
2. more than 160 test compounds with zero Ro5 [39] violations and
3. more than 120 test compounds free of any undesirable structural alerts [40]

These rules were modified and made stricter for PBDS2 plate selection as follows (see Experimental section for details):

1. remove plates with very low numbers (<200) and penalize plates with low numbers (<300) of unique compounds passing the GDRS filters;
2. remove plates with very high numbers (>300) and penalize plates with high numbers (>160) of total Ro5 violations, summed up across the plate;
3. remove plates with very high numbers (>100) and penalize plates with high numbers (>30) of compounds failing the stricter structural filters [35, 40];
4. give combinatorial plates an initial advantage in the selection process but remove this advantage in the final optimization iterations to achieve convergence (see below);
5. remove all plates already in PBDS in order to avoid plate depletion issues [41].

Experience from constructing the PBDS subset [35], which was very successful in practice, had shown that plates in violation of the Ro40 which were severely penalized (their assigned random number received a penalty of $+1.0$) in the seeding process never got selected. Therefore, we felt confident that we could exclude unacceptable plates completely without loss of important chemical space.

In addition, we now applied moderate penalties to less desirable plates, according to stricter rules, in order to better prioritize the property space in PBDS2. A moderate penalty of 0.1 was added to the random number assigned, ensuring that these plates would not be in the top 10 % of plates in the random order. This was justified as any critical chemical space lost could be replaced through the later cherry-picked portion of the new PBDS2 selection process (see below). A similar moderate advantage of $-0.1$ was initially given to library plates to allow them a higher chance of selection.

**Fig. 1:**
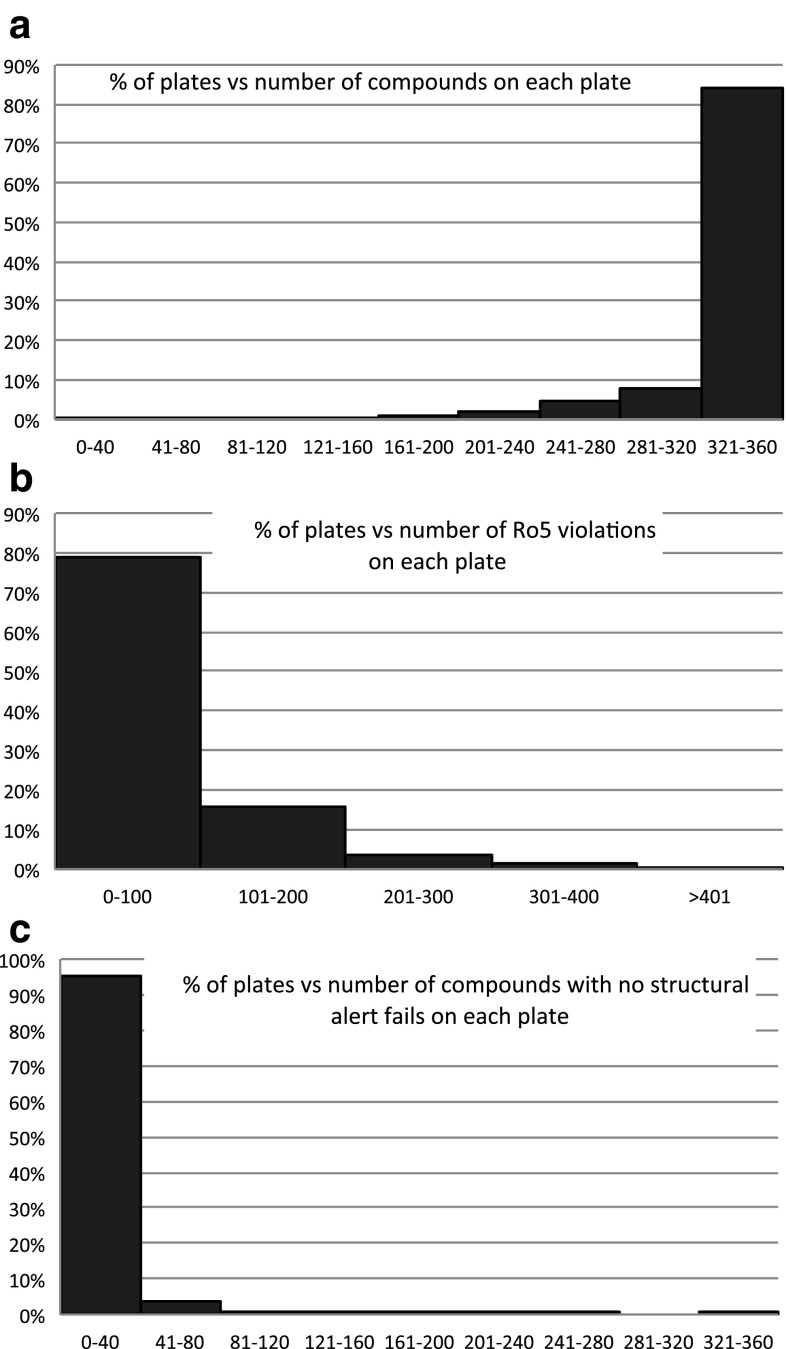
**a** Histogram of the number of screening plates in the Pfizer Screening File containing 0 to 40, 41 to 80, 81 to 120, etc. unique compounds per 384-well plate. The maximal number of test wells per plate is 360, as 24 wells are reserved for controls. **b** Histogram of the number of plates containing a total of 0 to 100, 101 to 200, etc. Ro5 violations per plate: note that any given compound on a plate could have more than one violation. **c** Histogram of plates containing 0 to 40, 41 to 80, etc. compounds per plate failing the stricter structural filters and thus being undesirable to a medicinal chemist. All data for Pfizer screening file as of 4th Quarter 2008.

Figure 1a shows a histogram of the number of unique compounds on each 384-well plate in the screening collection in November 2008. 98.5 % of the screening plates contain 200 or more compounds on and would therefore not be removed by the first rule of the PBDS2 plate selection process. Figure 1b shows the number of plates with 0 to 100, 101 to 200, etc. total Ro5 violations. Only 2 % of the plates have at least 300 Ro5 violations associated with them (maximum possible number of violations per $\hbox {plate} = 360 \times 4 = \hbox {1440}$) and would therefore be removed by the second rule. In addition, Figure 1c shows the number of compounds on each plate that fail the stricter structural filters [35, 40]. This demonstrates that the vast majority of plates would also not be removed by rule 3 of the PBDS2 plate selection rules.

The process outlined above was used to remove or penalize plates. Removed plates were not considered again during the selection process. Penalized plates got a penalty in the random seeding of plate orders that ensured they were only selected if the same region of space could not be adequately covered by non-penalized plates: see Experimental section for more details.

#### BCUT chemical space for PBDS2 plate selection

We elected to use BCUTs as implemented in DiverseSolutions$^{\textregistered }$ [42] to define the chemical space of the screening file in preference to fingerprints or molecular framework approaches, as this had worked very well for the design of the original PBDS [35]. We chose the cell-based BCUT approach [38] that provides both inter-compound distances and absolute positions in defined chemistry space and enables effectively all diversity tasks. In designing PBDS2, a five-dimensional (5D) chemistry space was used (a 6D space was used for PBDS) as it best represented compound diversity and yielded the most uniform distribution of compounds in the BCUT space. At the same time, we increased the number of bins in the 5D space from the 8 used in the PBDS, to 12. Based on an analysis of diversity distributions for each of the BCUT metrics, we found that including the BCUT metric of hydrogen bond acceptor would introduce both many empty cells and many highly dense cells and therefore was considered as an improper metric to use for describing the diversity of the compound collection. Removing this less-relevant metric and increasing the number of bins resulted in a similar total number of BCUT cells describing the chemical space of the screening file (248,832 vs. 262,144 in the original PBDS analysis) but an increase in the percentage of occupied cells and a reduction in the number of cells occupied by high numbers of compounds, thus producing a more evenly distributed chemical space. This lowers the randomness of selection within highly occupied cells, as well as the occurrence of relatively diverse compounds randomly occupying the same cell. Overall, this resulted in a more even and diverse subset.

As for the original PBDS, we chose to define the target chemical space of the screening file to be covered as those BCUT cells with an occupancy of 10 compounds or more. This was in order to avoid optimization of the PBDS2 screening subset on compounds with unusual structures in low-occupancy cells [35].

In the original PBDS, our target was to achieve a double coverage of >95 % of the BCUT cells with 10 or more compounds in them, i.e. the PBDS would contain at least two compounds from >95 % of the BCUT cells with an occupancy of $\ge 10$ compounds. Double coverage of the BCUT cells was selected to mitigate against two factors: (i) HTS is a noisy detector and could generate a false-negative result and (ii) there is inherently still a diversity of compounds within each cell. For PBDS2 in contrast to PBDS, we wanted to achieve a double coverage of 100 % of the cells with an occupancy $\ge$10 compounds, in order to ensure that the set had an improved representation of the chemical space of the file as a whole. This improvement is difficult to achieve using plate-based selection alone, as the iterations achieve a diminishing return at high percentage cell coverage, so we investigated a hybrid approach with a plate-based subset augmented by cherry-picking of individual compounds in order to achieve complete double coverage of BCUT space.

#### Pfizer screening file: library and non-library compounds

**Fig. 2:**
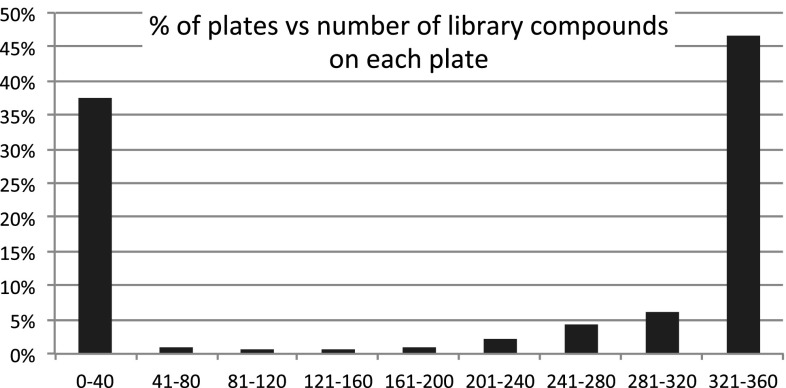
Percentage of screening plates against the binned number (0 to 40, 41 to 80, etc.) of library compounds on each plate in the filtered Pfizer screening file as of November 2008. The polarization of plates into those derived from library chemistry and those not is clear

The original 2006 file analysis ahead of the creation of PBDS showed that the file was polarized between plates that contained mostly library chemistry compounds with low overall chemical space diversity, together with plates containing mostly non-library compounds with much higher chemical space diversity [35]. This plate-type distribution has important consequences for the selection of a plate-based diverse subset, as the library plates tend to have low BCUT space coverage relative to non-library plates and therefore tend not to be selected for a diversity-based subset. We therefore re-analysed the enlarged file in November 2008. This analysis (Fig. 2) showed that whilst the proportion of library compound-containing plates had grown (*ca* 60 % of plates in 2008 containing in excess of 160 library compounds versus *ca* 54 % in 2006), the screening file was still very largely polarized into plates containing mostly library compounds and those containing mostly non-library (singleton or array) compounds.

#### Quality of compounds vs activity

A recent publication [43] describes a selection process using plate-based biological activity in previous screening campaigns as the main driver to derive an optimal plate-based selection for screening purposes. Prior to the PBDS2 work, we had developed a scoring scheme for Pfizer virtual libraries and for external compound collections to distinguish promising collections and libraries from potentially less successful ones. The scoring scheme was based on mapping a large number of individual compounds into chemical space and statistically determining areas of enrichment in biological activity in the chemical space. The scheme worked for any other property associated with individual compounds.

Mapping the scores for biological activity of multiple, external, commercial compound collections available in 2008 versus the score for Ro5 compliance generated an unexpected result [44].

**Fig. 3:**
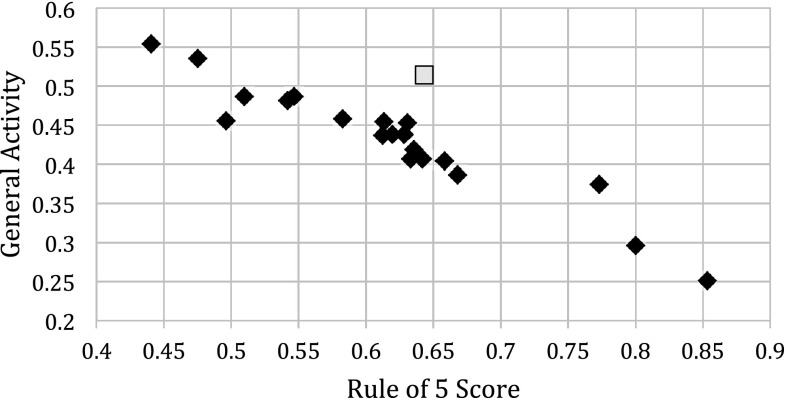
A plot of General Activity vs Rule of 5 scores for multiple, diverse screening sets [44]. The closed black diamonds are for external company or organization libraries. The open yellow square is for known drugs. See Experimental section for definitions and calculations of General Activity and Rule of 5 scores

We found a near-perfect linear inverse correlation between good scores for activity and good scores for Ro5 space for all compound collections (Fig. 3). Similar trends were found for biological activity score against clogP, molecular weight and molecular solubility. The only true outlier to all these trends was the drug collection, implying that drugs have an enhanced combination of biological activity and properties. With hindsight, this result should not have been surprising: the process of going from hit to lead and from lead to drug will inevitably discard compounds with inferior activity/property combinations. In addition, the difficulty of combining good physical properties with excellent activity in a drug molecule is well known [45–48].

It is unlikely that a simple algorithm will be able to drive compound SAR directly into drug space and thereby break through the above correlation. It therefore becomes a question of strategy. Is it better to start drug discovery projects with compounds with good properties and improve on their activity, or start with very active compounds and improve their properties?

Our strategy was based on the experience that it is easier to increase potency than it is to build in good properties. We also recognized that combinatorial chemistry offers a more efficient follow-up mechanism. We therefore deliberately did not use activity data during the selection process as this would lead us away from the Ro5 space and the combinatorial chemistry space. This strategy was consistent with our focus on the discovery of lead-like compounds [49, 50].

#### PBDS2 Plate selection for optimal BCUT space coverage

The iterative plate selection algorithm used for PBDS2 plate selection was developed from the methodology [35] used for the original PBDS. An initially random plate order was chosen to avoid false minima in the selection of the final plate order. The plate order was then optimized in an iterative process that used the number of additional BCUT cells covered by new plates as the fitness function (see Experimental section for further details). The new developments made for this implementation relative to that used for PBDS were as follows. The necessity to select equal numbers of library and non-library plates embedded into the PBDS plate selection algorithm was removed for PBDS2. Library chemistry plates were initially given an advantage in the selection process to give them a higher chance to be picked. However, this preferential treatment of library plates was removed after 80 % of iterations were completed to allow convergence based purely on optimal BCUT space coverage. This preferred treatment of combinatorial plates was applied to ensure that high-quality combinatorial plates would have the best chance to be selected in preference to non-combinatorial plates covering a similar BCUT chemical space, as hit follow-up of library compounds is facile. At the same time, removing the previous restrictions on a fixed ratio between combinatorial and non-combinatorial plates ensured that non-combinatorial plates would be selected once combinatorial plates no longer covered the unmapped space in an economical way.

**Fig. 4:**
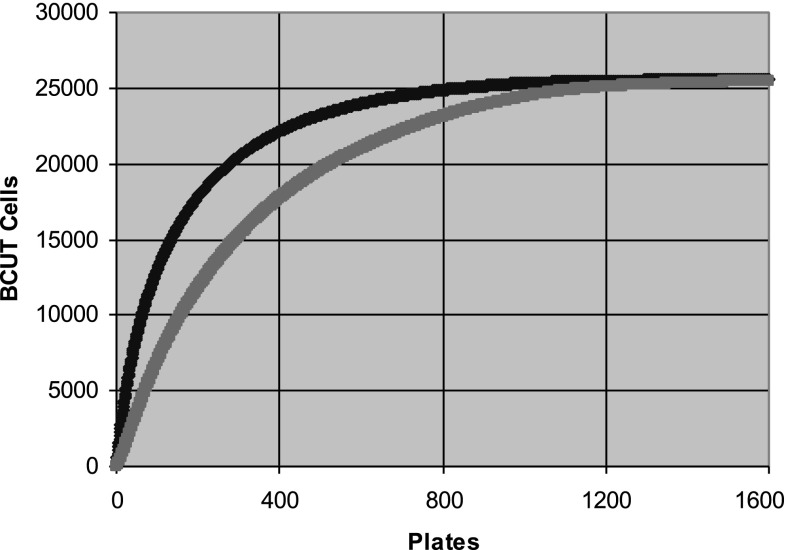
A plot of the number of BCUT cells in the Pfizer screening file covered for the first time (single coverage, upper dark blue line) and second time (double coverage, *lower*, *pink line*), plotted against the number of 384-well screening plates selected. Note the convergence of the iterative plate selection algorithm after the selection of 1200 plates. (Color figure online)

The PBDS2 plate selection achieved a single coverage of *ca* 97 % and >99 % and a double coverage of *ca* 90 % and >95 % of target BCUT cells from the selection of the best 800 and 1000 plates, respectively (Fig. 4), a considerable improvement upon the original PBDS [35], where 1200 plates were required to provide a double coverage of 95.4 % of the same target BCUT cells of a smaller file.

In order to achieve a double coverage of 100 % of the target BCUT space efficiently, and especially to ensure that PBDS2 had good coverage of library compounds, we added a cherry-picked element to PBDS2. A variety of targeted cherry-pick approaches were investigated, including the selection of representative compounds from clusters inside unrepresented BCUT cells. In this trial approach, the “fill-in” approach of DiverseSolutions$^{\textregistered }$ [42] was used for the cherry-picking. However, no targeted method worked as well as a fully random cherry-pick of compounds from the portion of the screening file that was not represented in the plate-pick (see Supplementary Fig. 1 and Supplementary Tables 1 and 2 for further information).

### In silico validation of plate-based diversity subset generation 2 (PBDS2) approaches

The PBDS2 subsetting approach was validated using a retrospective in silico analysis of hit $$ {series}$$ retrieval against ca 68,000 confirmed actives with $$\mathrm{IC}_{50}$$ values of <10  $$\upmu \hbox {M}$$ from 77 recent singleton HTSs run in the period prior to PBDS2 design at Pfizer. These 77 HTSs were executed against a broad range of targets in both biochemical and cell-based assays. The in silico series retrieval testing was performed against three different scenarios, each involving 400,000 selected compounds: (i) 100 % plate-based set, (ii) 75 % plate-based/25 % cherry-picked set and (iii) a 50 % plate-based and 50 % cherry-picked set. In the latter two cases, the cherry-pick was done randomly against all compounds not yet selected by the preceding iterative plate selection process. In each case, the 400,000 compounds represented 11 % of the full Pfizer file at that time (Table 1).

**Table 1:** The composition of the Pfizer screening file in fourth quarter 2008 prior to the construction of PBDS2

File	Number of plates	Number of compound IDs	Number of SMILES	Number of unique SMILES	Number passing GDRS filters
Singleton 384 well	8998	3,239,280	3,041,481	3,040,455	2,848,710
PBDS [35]	1200	432,000	422,704	422,685	414,460
Total	10,198	3,671,280	3,664,185	3,463,140	3,263,170

**Fig. 5:**
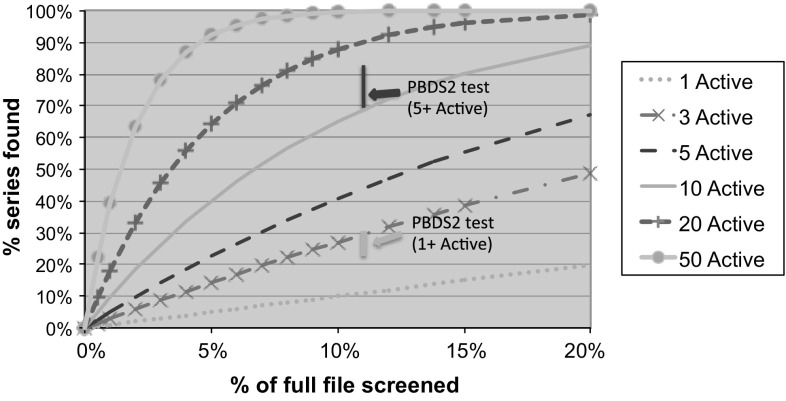
Calculation of the number of HTS hit series that would be expected to be found by random screening of between 0 and 20 % of the screening file for a series of cluster sizes of between 1 and 50 compounds (*curves*). Superimposed on these graphs are the calculated performances (shown by *vertical lines*) of a PBDS2 subset of 400,000 compounds when tested in silico against 68,000 HTS hits with $\mathrm{IC}_{50} < 10\,\upmu \hbox {M}$ from 77 recent HTSs and covering a range of cluster sizes, based on different molecular similarity criteria. The test PBDS2 subset in this case was constructed 50 % from plate-based selection followed by 50 % from random cherry-picking from those compounds not yet selected by the plate-pick. The vertical lines show the percentage of series that this PBDS2 construct was calculated to retrieve for a series of at least 1 active (*light blue*) and for a series of at least 5 actives (*blue*). See Supplementary Fig. 2 and Supplementary Tables 1 and 2 for more information. (Color figure online)

Figure 5 shows two elements: Firstly, a series of curves were calculated using Belief Theory methods, as applied previously in our molecular redundancy work [17], to calculate the percentage of series that should be found on a probabilistic basis, taking into account cluster size, by randomly screening different sized subsets of the Pfizer screening file. As would be expected, the probability of series retrieval rises sharply with the size of each series/cluster, calculated here for a series of between 1 and 50 compounds. Secondly, superimposed on these six curves are two vertical lines showing the series retrievals for a test PBDS2 subset of 400,000 compounds composed 50 % of plate-based and 50 % of cherry-picked compounds (random cherry-picking of compounds not selected in the plate-pick). The 50/50 plate-based/cherry-picked composition of this test PBDS2 set was used to test the success of the overall concept, with the final PBDS2 composition being determined later (see below). Two ranges of expected percentage series retrieval are given: one for a scenario where all series have at least 5 active compounds and the other for a scenario where the series can have any size from a singleton active upwards. In each case, the range of percent series found is based on calculations using single-linkage clustering with Tanimoto similarity boundaries of 0.6, 0.7, 0.8 and 0.9 (Supplementary Table 2). It is clear that for this test PBDS2 design of 400,000 compounds (11 % of the screening file), that series retrieval for all series (including singletons) is between 23 and 30 %. However, for larger series of 5 or more compounds, the in silico calculated series retrieval rises to between 69 and 83 %; an approximately 6- to 7-fold enhancement relative to random screening of 11 % of the whole file.

We then analysed different ratios of plate-picked to cherry-picked compounds for the final design of PBDS2. Table 2 below shows that the retrieval of all series varied only slightly from 33 to 31 % and then to 27 % as the plate selection portion is reduced from 100 to 75 % and then to 50 % of the subset. However, the coverage of larger series (5 or more compounds per series) increases more significantly from 67 % to 74 % and then 75 %, respectively, and the percentage of libraries covered increases dramatically from 17 to 100 % and 100 %, respectively.

**Table 2:** An overview of the calculated series retrieval and fold efficiency, and the actual library coverage of three PBDS2 designs of 400,000 compounds with 100, 75 and 50 % of the subsets chosen by iterative plate selection (see above) and the balance chosen by random cherry-picking from the remaining compounds not selected in the plate selection process

Percentage of plate-based/cherry-picked set in test PBDS2s	100/0	75/25	50/50
% Of total series found	33	31	27
% Of larger series found ($\ge$5 actives)	67	74	75
% Of libraries covered at $\ge$level of GDRS2, a 2nd generation cherry-picked subset [28] of 150,000 compounds used by therapeutic area teams	17	100	100
Fold efficiency over full file singleton for finding larger series	6.1	6.7	6.8

The series retrieval is calculated as described previously against 68,000 active compounds with $\mathrm{IC}_{50} \le 10\,\upmu \hbox {M}$, found in 77 recent Pfizer HTSs

**Fig. 6:**
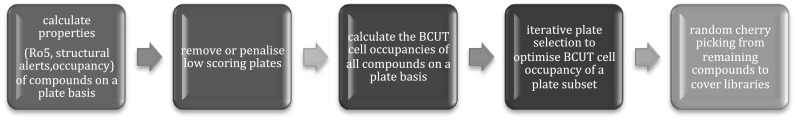
A flow chart depicting the five main stages of the PBDS2 plate selection process

The final design for the PBDS2 was chosen to be 75 % plate-picked and 25 % random cherry-picked from the remaining compounds not selected from the plate-pick. The final selection comprised 1200 plates: 900 from the plate-pick and 300 from the cherry-pick. This design was chosen for the following reasons: (i) the library coverage was dramatically improved over the subset designed with 100 % plate-picking; (ii) the efficiency of finding all series was only slightly reduced relative to the 100 % plate-picked design, (iii) the efficiency of finding larger series was superior to that of the 100 % plate-picked design and almost equivalent to that of the 50/50 plate-picked/cherry-picked design and (iv) the significant reduction in material management resources required to pick ca 108,000 compounds (25 %) relative to 216,000 (50 % of the set). The selected PBDS2 set had a total of 308,833 compounds from plate selection and 108,000 compounds from cherry-picking, totalling 416,833 compounds that comprised 11.4 % of the screening file. The flow chart (Fig. 6) provides a simplified overview of the entire PBDS2 plate selection process.

### Properties of the plate-based diversity subset generation 2 (PBDS2)

#### Target chemical space coverage of the selected PBDS2 plates

The selection process resulted in

1. 100 % double coverage of the BCUT space of the 2009 file (Table 3),
2. a representation of non-library compounds vs library compounds of approximate 2:1, compared to a forced 1:1 representation in the original PBDS [35],
3. 100 % coverage of all libraries due to the cherry-picked portion of the selection and
4. a significantly increased hit rate compared to a random selection of screening compounds (see Fig. 5 in Sect. 3.2 above).

**Table 3:** An analysis of the cell occupancies in PBDS2

PBDS2 BCUT cell analysis	
BCUT cell compound population	Number of BCUT cells
1–20	31,727
21–40	2584
41–60	948
61–80	517
81–100	307
101–120	195
121–140	124
141–160	80
161–180	59
181–200	33
201–220	32
221–240	22
241–260	19
261–280	8
281–300	6
301–320	6
321–340	2
341–360	1
361–380	0
381–400	1
Total number of occupied cells	36,671
Average occupied cell population	11.2
Standard deviation of average occupied cell population	7.2

#### Property distributions of the compounds selected in PBDS2

The 1200 plates of the PBDS2 that were selected were analysed to ensure that the distribution of molecular and calculated properties of the compounds in the subset was satisfactory and at least comparable with those of the original PBDS [35], especially for cLogP where high values had caused the majority of the original PBDS Rule of 5 failures. The average cLogP and molecular weights in PBDS2 were 3.02 and 367 Da against the values of 3.27 and 380 Da, respectively, for the original PBDS. The percentage of compounds with 0 and 1 Rule of 5 failures were 85.9 and 11.5 % in PBDS2 compared with 87 % and 11 %, respectively, in the original PBDS. The percentages of neutrals, bases and acids in PBDS2 was 60.6, 30.5 and 8.9 %, respectively. Further information is provided in Supplementary Fig. 2.

### Hit and series discovery using PBDS2 in live screening campaigns

The PBDS2 subset was utilized in more than 25 HTS campaigns over the subsequent 3-year period at which time a new version of the subset was created to avoid plate depletion caused by unanticipated non-HTS use of the initial segment of the PBDS2 [40]. The HTS campaigns included both cell-based and biochemical approaches, covered a broad range of target types including kinases, G-protein-coupled receptors (GPCRs), ion channels, transcription factors, epigenetic factors, a variety of enzymes and protein–protein interactions, and included phenotypic approaches measuring known, clinically relevant endpoints. The GPCR targets were addressed via multiple approaches including both binding and functional screens to identify a variety of agonists, inverse agonists and allosteric modulators.

**Fig. 7:**
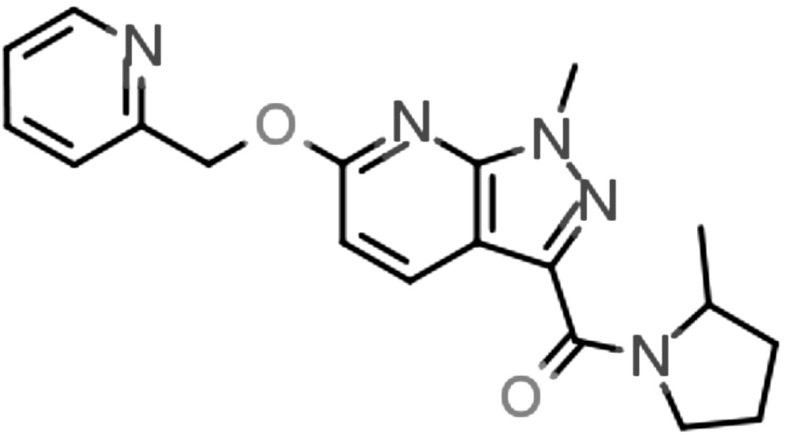
Compound 5 above from Zhang et al. [51] was an early lead developed from a PBDS2 subset hit and has mGluR5 $\hbox {Ki} = 6.60\hbox { nM}$, Geometric mean, $n \ge 3$ measurements in HEK-293FT cells expressing human mGLUR5 using [${}^{3}\hbox {H}$]MPEPy ([${}^{3}\hbox {H}$]3-methoxy-5-pyridin-2-ylethynylpyridine); mGLUR5 $\mathrm{IC}_{50}= 14.5\hbox { nM}$ Geometric mean, $n \ge 3$ measurements in HEK-293 cells expressing rat mGluR5 using fluorimetric imaging plate reader (FLIPR)

A screen of the PBDS2 subset was run at $10\,\upmu \hbox {M}$ compound concentration using transfected HEK293 cells and a calcium flux format to search for negative allosteric modulators of the metabotropic glutamate receptor 5 (mGluR5). This campaign successfully identified multiple active series including a novel series of pyrazolopyrimidines that have been reported and described in detail [51] (Fig. 7).

Additional GPCR screens in alternate formats were also successful in identifying valid actives. A screen of the PBDS2 at $10\,\upmu \hbox {M}$ compound concentration for agonists of GPR119 using a cAMP readout yielded a 0.8 % primary hit rate at >40 % activity. Subsequent screening of the parental cell was used to eliminate false positives and a beta-arrestin screen was also used in hit triage. The known lead matter was active and 4 novel series were identified for chemistry pursuit until the project was terminated for strategic reasons.

A second agonist screen for compounds modulating GPR120 was run at $20\,\upmu \hbox {M}$ compound concentration in a beta-arrestin format and yielded a 1 % primary hit rate at >20 % activity. Non-selective matter was eliminated using an alternate GPCR target. The screen identified previously known chemical matter but the project team did not pursue these compounds as they were seeking material with greater brain penetration. A total of 5 novel series were also identified, 3 of which were pursued into hit-to-lead phase, until the program was terminated for strategic reasons.

A screen of the PBDS2 was undertaken for inhibitors of the serine hydrolase, monoacylglycerol lipase (MAGL), as potential therapeutics for neuroinflammatory disease. The screen used a previously described, biochemical assay [52]. Compounds were screened at $10\,\upmu \hbox {M}$ concentration and multiple lead series were identified including compounds that were demonstrated to have competitive covalent and competitive non-covalent mechanisms of action. The irreversible azetidine carbamates have been disclosed in a previous presentation [53]. Binding to the target protein has been confirmed through x-ray crystallography and the series has been pursued to advanced stages with active chemistry design and synthesis.

Multiple PBDS2 screens for epigenetic factor inhibitors using an alphascreen format have been plagued with false positives. Following one screen targeting the epigenetic reader, BRD4, elimination of many of these spurious compounds was achieved with a fluorescence polarization assay leaving approximately 100 true actives of interest. One of the primary hits from the PBDS2 subset demonstrated $\mathrm{IC}_{50} < 200\,\hbox {nM}$ in the polarization assay and subsequently displayed activity in cell-based assays of human whole blood and human peripheral blood mononuclear cells, indicating the ability of the library to identify matter that translates to more physiologically relevant systems. In a second example, targeting a lysine demethylase eraser protein, selected compounds from the 22,000 primary hits were triaged through a high-throughput mass spectrometry assay and approximately 780 compounds achieved >50 % activity at $10\,\upmu \hbox {M}$ concentration. A total of 604 compounds yielded $\hbox {IC}_{50} < 10\,\upmu \hbox {M}$, providing further examples of translation to orthogonal technologies.

In another program searching for activators of the TREK-1 potassium channel, a high-throughput, compressed format thallium flux assay ($9.1\,\upmu \hbox {M}$ per compound) yielded no series of interest for medicinal chemistry. As a result, the PBDS2 was screened, since the high-quality singleton collection enabled a higher screening concentration to be used ($22.4\,\upmu \hbox {M}$). This campaign successfully yielded a 0.5 % primary hit rate with multiple series, making the translation to electrophysiology screens.

One campaign for protein upregulation utilized a promoter-driven reporter readout in a disease-relevant cell line. The PBDS2 collection was screened yielding a 0.5 % primary hit rate for compounds demonstrating >30 % effect. These compounds were subsequently tested in an orthogonal screen confirming protein expression (Delfia format). Approximately 12 % of the initial hits confirmed in the secondary assay and more than 100 compounds were demonstrated to have $\mathrm{EC}_{50}$ values <10 $\upmu$M. From these compounds, a total of 38 were selected and used to seed hit expansion efforts from the full chemical file, generating an additional 10,000 compounds that were profiled in the reporter assay with a 7.5 % hit rate. A total of 128 of these hit expansion compounds had >15 % activity at $10\,\upmu \hbox {M}$ in the orthogonal assay and ultimately 13 compounds were validated by the project team. These compounds were ultimately not pursued into development since they were subsequently shown to be acting via transcriptional pathways that yielded undesirable adverse effects.

Not all screens were so successful; two campaigns for oncology indications screened against protein–protein interactions yielded no hits that were suitable for chemistry follow-up. One tested the PBDS2 subset and additional matter in both fluorescence polarization and alphascreen formats. The second also tested multiple chemical libraries with different variations of an Alphalisa readout. The druggability of both targets has been called into question. A third protein–protein interaction for oncology also failed to yield hits from a scintillation proximity assay that tested both the PBDS2 and a commercially available file. A legacy Wyeth screen for the same target in Delfia format also failed to produce validated hit matter. None of the 3 files appeared to contain any analogues of the only publically known series at the time.

## Discussion

### Need for a second-generation PBDS

PBDS had been successful as a default subset of 429,067 compounds for lead finding by singleton HTS since its introduction in Pfizer in 2006. By late 2008, a large number of new compounds had been added to the screening file from library synthesis, individual compound synthesis and compound and company purchases. There was therefore a need to update the PBDS to ensure that it remained representative of the chemical space of the enlarged screening file and a desire to improve upon its chemical space coverage. The PBDS design had included a fixed alternate selection of library and non-library plates in order to ensure that the valuable library chemistry that Pfizer had invested so much resource in was represented in the subset. However, because library plates tend to be rather non-diverse, this stipulation meant that the efficiency of chemical space coverage was non-optimal, as some of the library plates selected for PBDS were non-diverse and contained compounds that with new understanding, we now regarded as structurally redundant [17]. Re-plating the complete file was not an option, as it was far too large a material management task. We needed a new process that would select suitable representatives of these library compounds but avoid the selection of entire plates full of very similar compounds from one or more libraries. We thus abandoned the requirement for alternate selection of library and non-library plates for PBDS2 and moved to a new, hybrid selection approach.

### Selection of the screening plates for PBDS2

The double coverage of target BCUT space for PBDS2 relative to PBDS was much improved, as the requirement to select equal numbers of library and non-library plates was removed. As a consequence of this change, library plates with low additional BCUT space coverage tended not to get selected. We therefore experimented with the notion of supplementing the plate-picked portion of the subset with a cherry-picked portion that would serve to fill in the holes of BCUT space missed by the plate selection algorithm and, in particular, to ensure that every individual library in our screening file was represented in PBDS2. This was relatively easy to monitor and execute as all library compounds have a unique identifier specific to each different library design, as well as a unique compound identifier. An analysis of series retrieval statistics gave excellent results for a 75/25 % plate-pick/cherry-pick design (Table 2). The fold efficiency for the retrieval of larger series relative to full file screening increased from 6.1-fold to 6.7-fold in progressing from a 100 % plate-pick design to a 75 % plate-pick/25 % cherry-pick design. This large improvement in screening efficiency is attributed to the 75/25 % PBDS2 design being better able to retrieve larger series that include library compounds. In the 100 % plate-pick design, some library compounds may be either overrepresented and redundant, *e*.*g*. through selection of a plate dominated by a single library, or missing altogether due to non-selection of non-diverse library plates.

The final design was implemented with random cherry-picking from all of the compounds that had not been selected in the initial iterative plate-pick of 900, 384-well plates. Intuitively, we felt that a deterministic cherry-pick based on choosing compounds representative of clusters from the unpicked compounds should be a superior methodology to random selection. However, extensive in silico analysis of active series retrieval showed that random cherry-picking outperformed our clustering methods, in every configuration of cluster similarity and cluster size that we analysed (see Supplementary Fig. 1 and Supplementary Tables 1 and 2 for further data). We therefore implemented PBDS2 with 25 % random cherry-picking from those compounds in the screening file unselected following the iterative plate-pick.

### Performance of the PBDS2

PBDS2 replaced PBDS as the default singleton screening subset for Pfizer therapeutic area targets in July 2009. Over the next 3 years, the subset was successfully used in more than 25 HTS campaigns, against a very broad range of targets, some of which are presented in the Results section. Additionally, PBDS2 was used for more phenotypic approaches capturing broader mechanisms of action measuring known, clinically relevant endpoints. If actives were found in a PBDS2 screen, the follow-up process was generally as follows: (1) confirm the activity of the putative hits with repeat, single-concentration assays; (2) measure the $$\mathrm{IC}_{50}$$ values of confirmed hits; (3) perform ‘hit expansion’ around selected confirmed hits using similarity searching in the remainder of the screening file to locate compounds with similar structural features to those hits and then screening them and finally (4) initiate *de novo* synthesis projects to follow up promising hit series. Whilst the selection of $$ {plates}$$ for PBDS2 used the Rule of 5 as one quality measure for plates of compounds as a whole, the selection of $$\textit{confirmed hits}$$ for follow-up would normally require compounds to pass stricter requirements including the Rule of 4 (molecular weight < 400 Da; $$\hbox {cLogP} < 4.0$$, less than 4 hydrogen bond donors and less than 8 hydrogen bond acceptors). This is because it is well known that in progressing from hit to lead to drug candidate, that molecular weight and lipophilicity tend to increase and therefore the starting hits are preferentially selected to be of lower molecular weight and lipophilicity than that which is optimal for drugs. The performance of the PBDS2 was excellent, generating high-quality hit series that translated to physiologically relevant systems and formed the basis for medicinal chemistry design and synthesis [51, 53]. Demand for access to this newly prioritized plate-based diverse subset was so great that eventually the material management stocks, which had been designed to last for ten years, became depleted and a complementary subset approach PBDSx was subsequently introduced to mitigate this [41].

## Conclusions

Plate-based diversity subset (PBDS) screening was a novel approach when introduced into Pfizer in 2006. It is a radical new approach that changes HTS from random screening to a deterministic, chemical space-based paradigm. It quickly became the default method for singleton HTS in Pfizer Global R&D and was highly successful. The new, second-generation PBDS2 represents an important development in subset design as it enables the capture of value from relatively low-diversity library series, without resorting to forcing the selection of library plates, as had been done in the original PBDS implementation [35]. In addition, the hybrid design of PBDS2, with the majority of the subset derived from iterative plate-picking, but 25 % derived from random cherry-picking of the remaining, non-selected compounds, enables the facile updating of the subset over time. All that is required is that, at intervals, new plates are added to the subset by judicious cherry-picking of compounds occupying new regions of BCUT space, as the plates containing those compounds are registered into the screening file. Like its predecessor, PBDS2 became the default subset for singleton HTS in Pfizer Global R & D following its introduction in 2009, on the basis of its simplicity of construction and its 6.7-fold greater efficiency in large series retrieval relative to full file singleton screening. In addition to the overall concept of PBDS, we believe that the randomly initiated plate selection algorithm and the plate selection rules presented here will be of utility to many academic and industrial drug discovery groups as they create new subsets for HTS [30, 32, 54].

The success of PBDS [35] and then PBDS2 in Pfizer over many years [51, 53] and the parallel success of the Novartis methods [36, 37] give us confidence that this approach will be of value for HTS of large screening files for many years to come. Indeed, a new approach to the plate selection algorithm has recently emerged from Novartis, where plates are selected principally according to the biological target diversity profile of the compounds on the plates [43]. A new variant has also emerged from Pfizer, where, due to increased use of PBDS2 and differential plate depletion, a novel methodology for the construction of several equivalent PBDSs was developed [41]. We look forward to further developments to improve drug discovery efficiency in the future.

## Experimental

### Dataset

The file analysis was performed on the Pfizer Screening File containing 10,198 singleton screening plates available for experimental screening from Pfizer Material Management as of autumn 2008. Each 384-well screening plate contained up to 360 test wells and 24 control positions. Information for each individual plate was in the form of compound identifiers mapped to well positions. This information was transformed into structure information using the Pfizer internal compound database. Batch information was ignored at this stage and only the desalted compound structure was used.

The screening plates came from multiple compound sources: Legacy Pfizer (including Warner Lambert), Pfizer library chemistry (File Enrichment, external, combinatorial compounds), Pfizer parallel medicinal chemistry (PMC, internal, combinatorial and non-combinatorial [33]) and legacy Pharmacia [17].

### Property calculations

Computational work on the dataset was carried out using Pipeline Pilot$^{\textregistered }$ from SciTegic/Accelrys [55]. File filters were applied as previously described [35]. Compound identities and duplication were assessed using the simplified molecular-input line-entry system or SMILES from Daylight Chemical Information Systems Inc [56]. Compounds with identical salt-free canonical SMILES were classified as duplicates. Rule of 5 properties were based on internal code with clogP values based on the BioByte code from Pomona College [57]. The chemical space occupancy of compounds on the screening plates was calculated using the BCUT methodology from DiverseSolutions$^{\textregistered }$, supplied by Tripos Inc [42], as previously described [35] but with modification; see below.

### Series definitions

Compound clustering was performed using Barnard 1052 fingerprints from Barnard Chemical Information Systems Ltd (30 Kiveton Lane, Todwick, Sheffield UK) and Tanimoto similarity metrics calculated using Daylight [56] fingerprint comparison tools. Each compound was assigned to a cluster if it had a Tanimoto similarity above a given threshold (0.6, 0.7, 0.8 or 0.9) to at least one other member in the cluster.

High Tanimoto values result in a large number of singleton compounds, while low Tanimoto values reduce significantly the number of singleton and result in larger clusters. Both descriptions have advantages and disadvantages and therefore the analysis was performed across the range of different similarity values given above.

### Chemical space definition

In designing PBDS2, a five-dimensional (5D) chemistry space was defined for our corporate collection using the “auto-choose” algorithm in DiverseSolutions$^{\textregistered }$ [42]. This chemistry space is different from the 6D space used for the original PBDS (we removed the BCUT metric of hydrogen bond acceptor), but gives a more uniform distribution of compounds in the BCUT space. At the same time, we increased the number of bins in the BCUT space from 8, as used in the PBDS, to 12 for PBDS2.

### General activity and Rule of 5 (Ro5) score definitions

The activity score and the Ro5 score methodologies were developed to compare and evaluate external compound datasets without the need to exchange structural data. BCUT space was used to describe the chemical space. The first step divided the chemical space into small bins – the individual BCUT cells. A background set was mapped into this space to represent the sort of compounds produced in the pharmaceutical industry. The Pfizer HTS screening file was used for this purpose as the best available set.

A reference set representing compounds with a specific quality or property was then mapped into the same space. The quality of the reference set was measured with binary outputs such as yes/no for a property such as biological activity, solubility or Ro5 compliance at a set level. This allowed the calculation of a score for each individual bin, i.e. BCUT cell, representing the relative density of compounds in the reference set with a particular property compared to the background set. A scaling factor was used to map reference sets of different sizes.

A score for any other test set could then be generated by mapping each individual test compound with a specific property set to yes into the reference set and calculating the average score of all of the compounds in the test set. The scaling factor k is given by

$$\begin{aligned} k=\frac{N}{A}. \end{aligned}$$

The score for individual bin i is then given by

$$\begin{aligned} score_i =\frac{k\times A_i }{k\times A_i +N_i } \end{aligned}$$

...and the averaged score for the test set is given by

$$\begin{aligned} score_{test} =\frac{1}{B}\times \mathop \sum \limits _{i=1}^B score_i \times B_i, \end{aligned}$$

where *N* is the total number of compounds in background set, in this case, the Pfizer screening file; $\hbox { {N}}_{i}$ is the number of compounds of background set in cell *i*; $\hbox { {A}}$ is the total number of compounds in reference set; ${ {A}}_{i}$ is the number of compounds of the reference set in cell *i*; $\hbox { {B}}$ is the total number of compounds in test set; ${ {B}}_{i}$ is the number of compounds of test set in cell *i*.

The reference set used for the activity score was a set of ca. 60,000 known active compounds (activity <10 $\upmu$M) from the Pfizer Drug Store database. The scoring method works well with a comprehensive reference set with broad chemical space coverage.

The Ro5 score was calculated by exactly analogous methods but using Ro5 compliance instead of biological activity as the property. Compliance was defined as complying with all of the Ro5 rules. This differs from the original Ro5 that allowed a single failure but was more in line with the stricter use of the rule in this work at Pfizer. Both the Ro5 score and the general activity score are scaled relative to the background file.

### PBDS2 plate selection

The PBDS2 plate selection used an iterative process with the following workflow implemented into Pipeline Pilot:

1. Individual plate statistics were derived prior to the selection process including the number of unique compounds on each plate, the total sum of Ro5 violations on each plate, the number of structural alert failures and the number of library compounds. Plates with >50 % library compounds were designated as library plates – these were given a bonus in the seeding process (see below).
2. All plates belonging to the original PBDS set [35] were excluded from the selection process. Plates of insufficient quality were removed prior to the selection stage (see “Results” section). Plates that had worse-than-average properties (see 1.2 as well) were given a penalty in the seeding process.
3. Each plate was allocated a random number prior to the first iteration, with a value between 0 and 1. Combinatorial plates received a bonus to this random number value $(-0.1)$, while plates with undesirable properties received a penalty $(+0.1)$.
4. Plates were ordered from low to high random number and it was determined how many new, unoccupied, or singly occupied BCUT cells each plate contributed. This value was highly order dependent and drove the use of the random ordering process.
5. The plates were then reordered according to the number of new BCUT cells filled for the first or second time.
6. New random numbers were then created for all plates. The top 50 plates from the first iteration retained their position, while all other plates were resorted according to their new random number, from low to high.
7. The number of newly filled BCUT cells was recalculated and all plates were again resorted according to this target function (number of new BCUT cells covered for the first or second time). This resorting included the original top 50 plates, which could now slowly drop in the ranking if fitter plates were present with greater additional BCUT space coverage.
8. The top 100 plates were retained after the second iteration and an additional 50 plates were retained after each subsequent iteration up to a maximum number of 800 retained plates.
9. All constraints in terms of plate score bonuses and penalties were removed after 50 iterations and a final 20 iterations were performed.

The total number of iterations required was determined through two test runs using 20 and 50 iterations in the absence of any constraints. The partly constrained process detailed above converged more slowly but to a better solution than the unconstrained processes.

## Electronic supplementary material

Below is the link to the electronic supplementary material.

Supplementary material 1 (pdf 373 KB)

## Abbreviations

cAMP: Cyclic adenosine monophosphate
cLogP: Calculated logarithm to base 10 of partition coefficient P of compound between n-octanol and water
ECFP4: SciTegic/Accelrys’ level 4 extended connectivity fingerprints
FE: File enrichment
GDRS: Global diverse representative subset
HTS: High-throughput screening
PBDS: Plate-based diversity subset
PBDS2: Plate-based diversity subset generation 2
PMC: Parallel medicinal chemistry
SAR: Structure–activity relationships
