# Supplementary material for: Plate-based diversity subset screening generation 2: an improved paradigm for high-throughput screening of large compound files
Source: Mol Divers. 2016 Sep 8;20(4):789–803. doi: 10.1007/s11030-016-9692-9 (PMC5055576; doi:10.1007/s11030-016-9692-9)
Supplement: Supplementary file 1 — Supplementary material 1 (pdf 373 KB) [file 11030_2016_9692_MOESM1_ESM.pdf]

Plate-Based Diversity Subset Screening Generation 2: An Improved Paradigm for High Throughput Screening of a Large Screening File: SUPPLEMENTARY MATERIALS

Andrew S. Bell, Joseph Bradley, Jeremy R. Everett, Jens Loesel, David McLoughlin, James Mills, Marie-Claire Peakman, Robert E. Sharp, Christine Williams and Hongyao Zhu

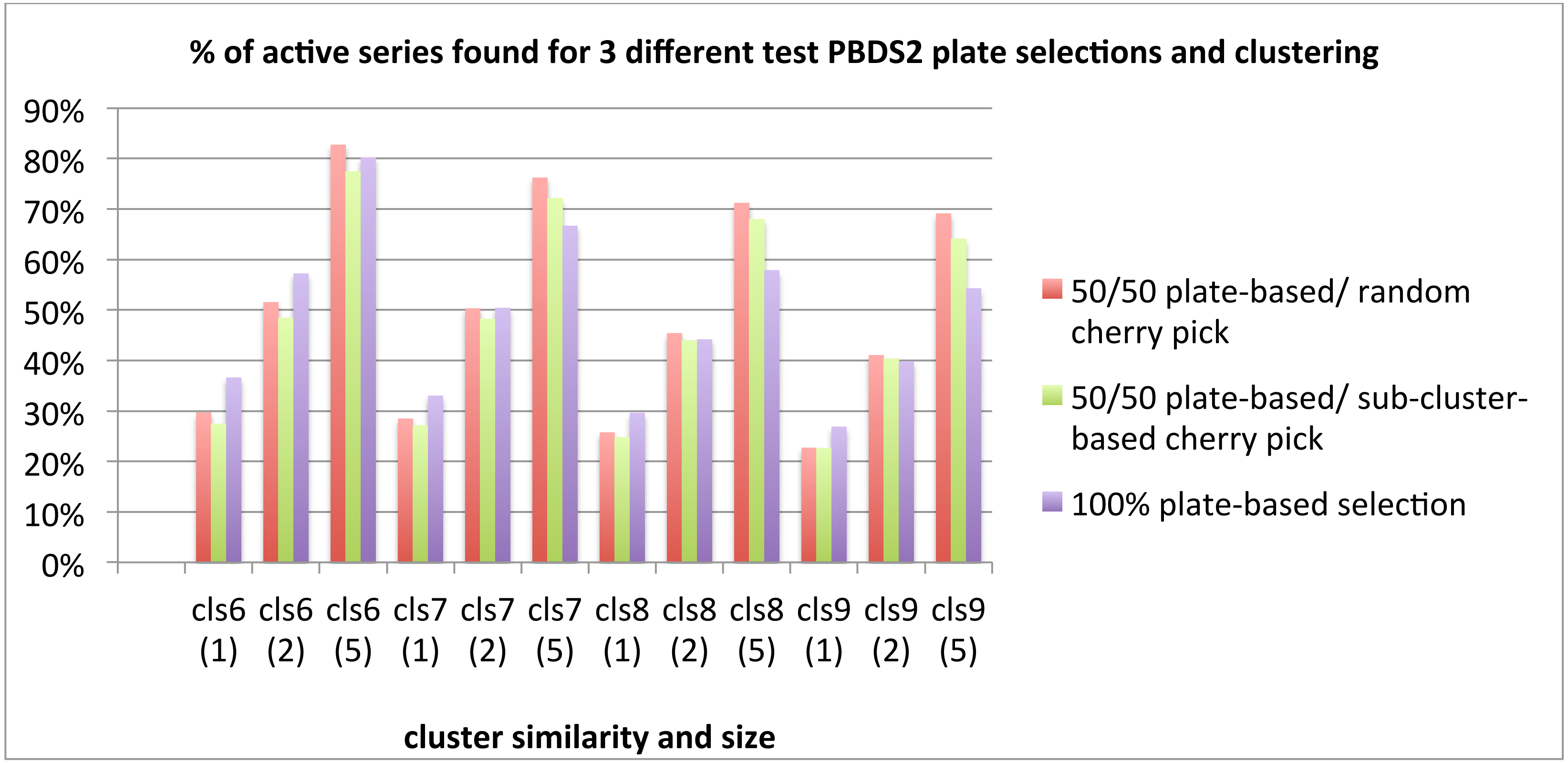

Supplementary Figure 1. A retrospective *in silico* analysis of the % of active series found against cluster similarity and size for three different test PBDS2 plate selections of 400,000 compounds: (i) 50% plate-based, 50% random cherry pick; (ii) 50% plate-based, 50% sub-cluster-based cherry pick and (iii) 100% plate-based selection. The analysis was based upon 68,000 IC_{50} values of $\leq$ 10 μM found in 77 representative cell-based and biochemical HTSs run in Pfizer in the period prior to PBDS2 design. Active series were clustered using Daylight software and with Tanimoto similarity coefficients of 0.6, 0.7, 0.8 and 0.9 for cls6, cls7, cls8 and cls9 respectively. The numbers in brackets on the x-axis represent the minimum cluster size i.e. (5) means all clusters with 5 or more active compounds in them. The raw data for this figure is found in Supplemental Tables 1 and 2 below. The cherry picks were performed for all compounds not selected in the initial 50% plate pick. In every case, random compound cherry picking outperformed cherry picking based on sub-clustering.

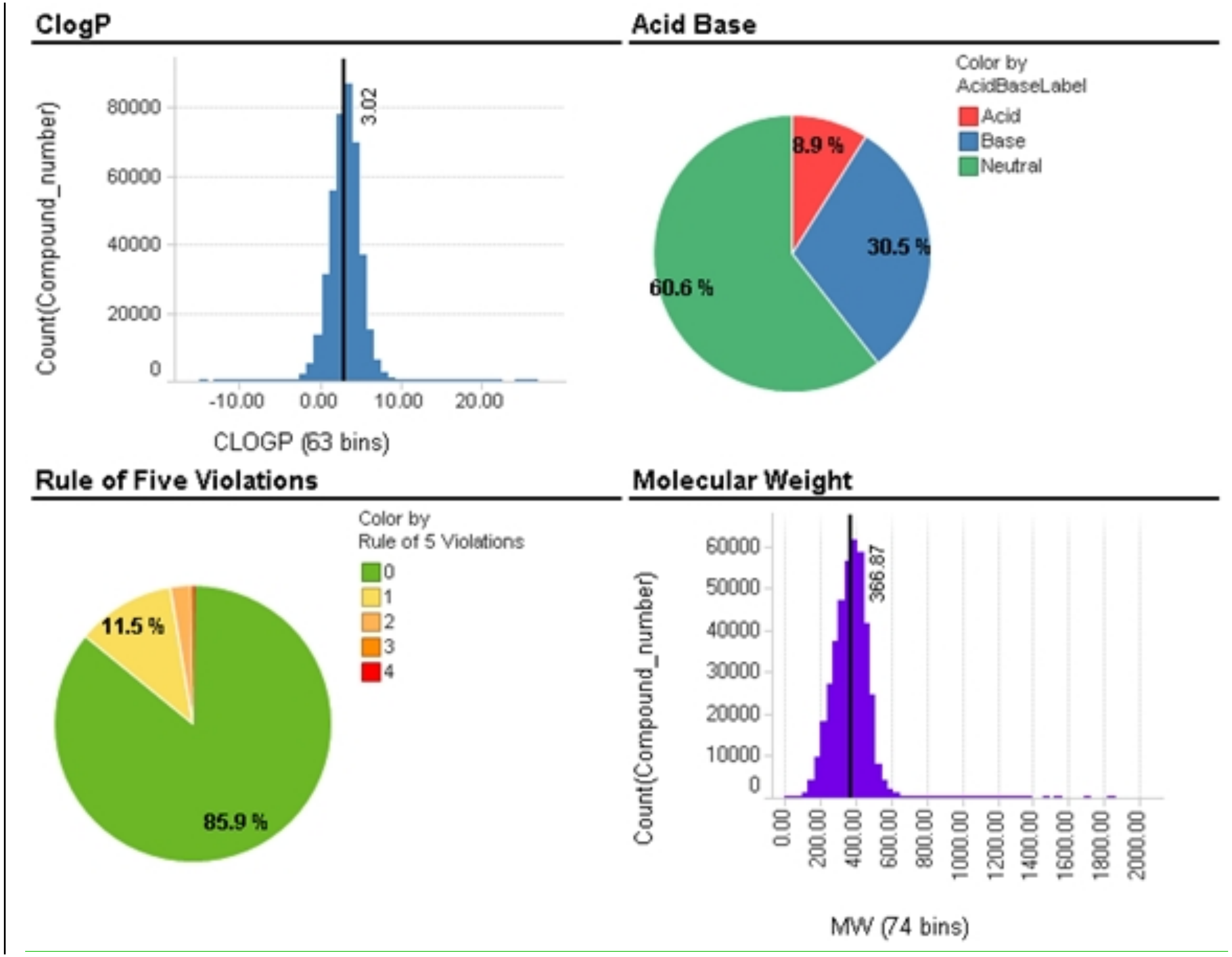

Supplementary Figure 2: The properties of compounds in PBDS2. Clockwise from top left: the distribution of clogP values; a pie chart of molecular class; the distribution of molecular weights; a pie chart of the % of PBDS2 compounds with 0 to 4 Rule of 5 violations.

	% of series found			number of series found			
cluster similarity and size	50/50 plate-based/ random cherry pick	50/50 plate-based/ sub-cluster-based cherry pick	100% plate-based selection	50% plate based, 50% random cherry pick	50% plate based 50% using sub clusters	100% plate based	total number of series
cls6 (1)	30%	27%	37%	2410	2229	2974	8120
cls6 (2)	51%	48%	57%	996	937	1107	1935
cls6 (5)	83%	77%	80%	422	395	409	510
cls7 (1)	28%	27%	33%	5538	5284	6425	19459
cls7 (2)	50%	48%	50%	2544	2440	2550	5062
cls7 (5)	76%	72%	67%	1209	1144	1057	1586
cls8 (1)	26%	25%	30%	7826	7552	9012	30478
cls8 (2)	45%	44%	44%	3106	3008	3024	6846
cls8 (5)	71%	68%	58%	1324	1263	1075	1859
cls9 (1)	23%	23%	27%	10083	10036	11919	44461
cls9 (2)	41%	40%	40%	2872	2826	2789	7001
cls9 (5)	69%	64%	54%	970	899	761	1403

Supplementary Table 1: A retrospective *in silico* analysis of the % and number of series found against cluster similarity and size for three different test PBDS2 plate selections of 400,000 compounds: (i) 50% plate-based, 50% random cherry pick; (ii) 50% plate-based, 50% sub-cluster-based cherry pick and (iii) 100% plate-based selection. The analysis was based upon 68,000 IC50 values of $\leq$ 10 μM found in 77 representative cell-based and biochemical HTSs run in Pfizer in the period prior to PBDS2 design. Active series were clustered using Daylight software and with Tanimoto similarity coefficients of 0.6, 0.7, 0.8 and 0.9 for cls6, cls7, cls8 and cls9 respectively. The numbers in brackets in column 1 represent the minimum cluster size i.e. (5) means all clusters with 5 or more active compounds in them.

clustering method	number of series, N	mean number of compounds in series	maximum number of compounds in series	median number of compounds in series	geometric mean of number of compounds in series	number of series retrieved by 50/50 plate-based/ random cherry pick	% series retrieval by 50/50 plate-based/ random cherry pick
cls6 (1)	8,120	8.4	4,878	1	1.4	2,410	30%
cls7 (1)	19,459	3.5	2,629	1	1.4	5,538	28%
cls8 (1)	30,478	2.3	494	1	1.3	7,826	26%
cls9 (1)	44,461	1.5	214	1	1.2	10,083	23%
cls6 (2)	1,935	32.2	4,878	3	3.9	996	51%
cls7 (2)	5,062	10.7	2,629	3	4.0	2,544	50%
cls8 (2)	6,846	6.6	494	3	3.6	3,106	45%
cls9 (2)	7,001	4.4	214	2	3.1	2,872	41%
cls6 (5)	510	115.2	4,878	9	15.3	422	83%
cls7 (5)	1,586	28.6	2,629	9	12.1	1,209	76%
cls8 (5)	1,859	17.5	494	9	11.0	1,324	71%
cls9 (5)	1,403	12.4	214	8	9.2	970	69%

Supplementary Table 2. An analysis of 68,000 actives with IC50s of $\leq$ 10 μM from 77 Pfizer HTSs, showing the number of series according to several different clustering methods using Daylight clustering with Tanimoto similarity cut-offs at 0.6, 0.7, 0.8 and 0.9: cls6, cls7, cls8 and cls9 respectively. The numbers in brackets in column 1 indicates the minimum series size considered in the calculations. For instance, the cls8 (5) row represents the analysis for clusters of 5 or more compounds with a Tanimoto similarity of > 0.8. Across all series, a test PBDS2 design with 50% plate selection, 50% random cherry-pick recovered between 23 and 30% of series (column 8). Across larger series of 5 or more compounds per series, the same design recovered between 69 and 83% of series depending on the clustering cut-off (column 8). See Figure 5 in the main paper for a visualization of this performance.
